# *In vitro* characterization of engineered red blood cells as viral traps against HIV-1 and SARS-CoV-2

**DOI:** 10.1016/j.omtm.2021.03.003

**Published:** 2021-03-10

**Authors:** Magnus A.G. Hoffmann, Collin Kieffer, Pamela J. Bjorkman

**Affiliations:** 1 Division of Biology and Biological Engineering, California Institute of Technology, Pasadena, CA 91125, USA

**Keywords:** Engineered red blood cells, anti-viral therapeutics, HIV-1, SARS-CoV-2

## Abstract

Engineered red blood cells (RBCs) expressing viral receptors could be used therapeutically as viral traps, as RBCs lack nuclei and other organelles required for viral replication. However, expression of viral receptors on RBCs is difficult to achieve since mature erythrocytes lack the cellular machinery to synthesize proteins. Herein, we show that the combination of a powerful erythroid-specific expression system and transgene codon optimization yields high expression levels of the HIV-1 receptors CD4 and CCR5, as well as a CD4-glycophorin A (CD4-GpA) fusion protein in erythroid progenitor cells, which efficiently differentiated into enucleated RBCs. HIV-1 efficiently entered RBCs that co-expressed CD4 and CCR5, but viral entry was not required for neutralization, as CD4 or CD4-GpA expression in the absence of CCR5 was sufficient to potently neutralize HIV-1 and prevent infection of CD4^+^ T cells *in vitro* due to the formation of high-avidity interactions with trimeric HIV-1 Env spikes on virions. To facilitate continuous large-scale production of RBC viral traps, we generated erythroblast cell lines stably expressing CD4-GpA or ACE2-GpA fusion proteins, which produced potent RBC viral traps against HIV-1 and SARS-CoV-2. Our *in vitro* results suggest that this approach warrants further investigation as a potential treatment against acute and chronic viral infections.

## Graphical abstract

**Figure:**
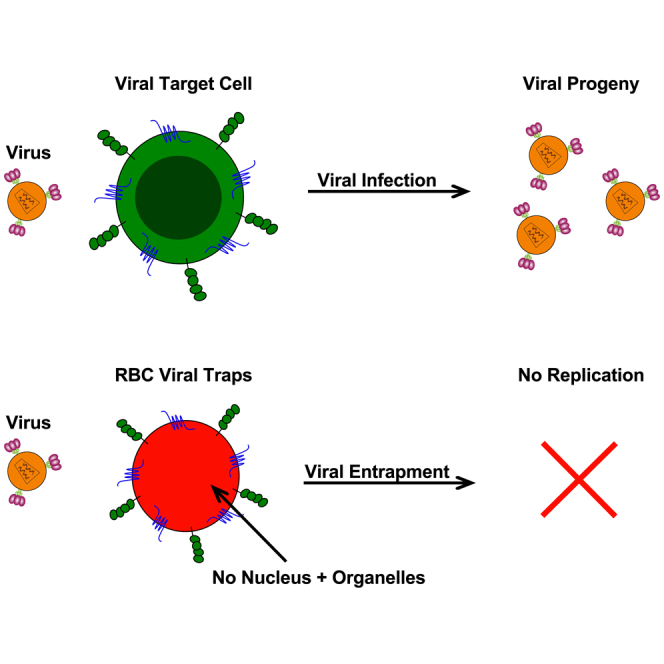

## Teaser

Hoffmann and colleagues genetically engineered erythroid progenitor cells to express HIV-1 receptors, which efficiently differentiated into enucleated red blood cells (RBCs) that potently neutralized HIV-1 *in vitro*. To facilitate large-scale production of engineered RBCs, erythroblast cell lines were generated, which continuously produced potent RBC viral traps against HIV-1 and SARS-CoV-2.

## Introduction

Red blood cells (RBCs) exhibit unique properties that can be exploited for therapeutic applications: they are the most abundant cell type, permeate all tissues, and have a lifespan of 120 days, making them attractive carriers for the delivery of therapeutic cargoes.^1^^,^^2^ Moreover, RBCs do not express major histocompatibility complex class I molecules,^3^ and thus therapeutic RBCs from type O-negative blood could be universally administered to patients.

Engineered RBCs have been proposed as ideal candidates for the design of viral traps, as they lack nuclei and other organelles required for viral replication.^4^, ^5^, ^6^, ^7^ Viruses could be lured into attaching to and infecting RBCs that present viral receptors, thereby leading the virus to a dead end and protecting viral target cells from infection. This approach has the potential to prevent viral escape, as viruses must retain the ability to bind their receptors. However, expression of viral receptors on RBCs is difficult to achieve since mature erythrocytes lack the cellular machinery to synthesize proteins. Hence, erythroid progenitor cells need to be genetically engineered to express the viral receptors and then be differentiated into enucleated RBCs. During the erythroid differentiation process, transgene expression is restricted through transcriptional silencing,^8^ translational control mechanisms,^9^ and degradation of proteins that are not normally present in RBCs.^10^

One strategy to overcome the latter problem is to generate chimeric proteins by fusing the extracellular domain of a non-erythroid protein to a protein that is abundantly expressed in RBCs, such as glycophorin A (GpA).^11^^,^^12^ However, this approach is limited to single-pass transmembrane proteins, prevents localization of viral receptors to their natural membrane subdomains, and might not achieve sufficiently high receptor levels to effectively entrap the virus. In the case of a potential HIV-1 therapeutic, additional strategies are required to generate RBC viral traps, as the HIV-1 receptors CD4 and CCR5 co-localize in nanoclusters within lipid rafts,^13^^,^^14^ and CCR5 is a G protein-coupled receptor (GPCR) with seven transmembrane domains.

In this study, we show that the combination of a powerful erythroid-specific expression system and transgene codon optimization yields high expression levels of the HIV-1 receptors CD4 and CCR5 on enucleated RBCs to generate viral traps that potently inhibit HIV-1 infection *in vitro*. We then applied these engineering strategies to generate erythroblast cell lines that can continuously produce potent RBC viral traps against HIV-1 and SARS-CoV-2.

## Results

### Enucleated RBCs express HIV-1 receptors

**Figure 1:**
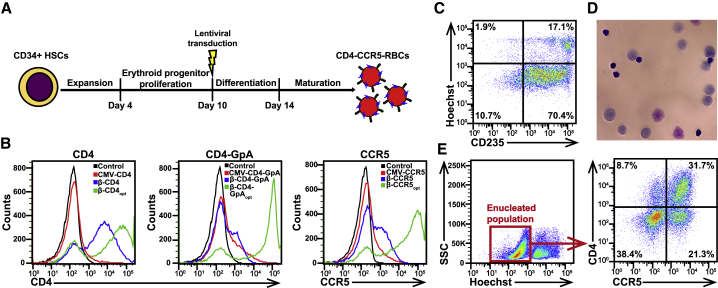
Engineered RBCs express HIV-1 receptors (A) Schematic illustrating the workflow for generating enucleated RBCs expressing HIV-1 receptors. (B) Flow cytometry analysis of CD4, CD4-GpA, and CCR5 expression on day 13 of differentiation comparing the CMV promoter (red), the β-globin promoter (blue), and the β-globin promoter in combination with codon optimization (green). (C) Quantification of enucleated CD4-CCR5-RBCs by flow cytometry. Enucleated RBCs expressed CD235 and did not stain for the nuclear dye Hoechst. (D) Image of CD4-CCR5-RBCs after May-Grünwald-Giemsa staining (original magnification, ×63). (E) CD4 and CCR5 expression on enucleated (Hoechst-negative) RBCs.

We used an *in vitro* differentiation protocol^12^ to differentiate human CD34^+^ hematopoietic stem cells (HSCs) into reticulocytes, an immature form of enucleated RBC that still contains ribosomal RNA (Figure 1A). At the end of the proliferation phase, erythroid progenitor cells were transduced using lentiviral vectors carrying CD4 or CCR5 transgenes by spinoculation (Figure 1A; Figure S1A). We also evaluated expression of a CD4-GpA fusion protein that contained the extracellular CD4 D1D2 domains fused to the N terminus of GpA, an abundantly expressed RBC protein. Three days post-transduction, transgene expression was evaluated by flow cytometry. Expression was low for all transgenes when the cytomegalovirus (CMV) promoter or alternative ubiquitous promoters were used (Figure 1B; Figure S1B). Surprisingly, CD4-GpA expressed only marginally better than CD4, suggesting that additional strategies are required to achieve robust expression of viral receptors on RBCs.

To evaluate whether transcriptional silencing can be prevented by using an erythroid-specific promoter, transgenes were subcloned into the CCL-βAS3-FB lentiviral vector,^15^ which contains regulatory elements that support the high expression levels of β-globin during erythroid development (vectors β-CD4, β-CD4-GpA, and β-CCR5) (Figure S1A). CD4 expression was greatly enhanced by this expression system, and CCR5 expression increased to a lesser extent, but CD4-GPA expression was not improved (Figure 1B).

We hypothesized that the limited availability of ribosomes and transfer RNAs potentially restricts transgene expression in differentiating erythroid cells. Transgene cDNA sequences were codon-optimized to generate β-CD4_opt_, β-CD4-GpA_opt_, and β-CCR5_opt_. For all transgenes, codon optimization drastically enhanced expression levels (Figure 1B). These results demonstrated that the combination of a powerful erythroid-specific promoter and transgene codon optimization yields high expression levels of HIV-1 receptors in erythroid cells.

Genetically engineered CD4^+^/CCR5^+^ erythroid progenitor cells differentiated efficiently into enucleated RBCs (Figure 1C). After differentiation, almost 90% of cells expressed GpA, of which >80% did not stain for Hoechst nuclear dye, suggesting that the majority of cells were enucleated RBCs (Figure 1C). May-Grünwald-Giemsa staining confirmed that most cells had lost their nuclei (Figure 1D). Approximately one-third of the enucleated RBCs expressed CD4 and CCR5 on their surface (Figure 1E) at levels comparable to Rev-A3R5 CD4^+^ T cells (Figure S2). Similar CD4^+^ T cell lines have been shown to express ∼10^5^ copies of CD4 and ∼10^3–^10^4^ copies of CCR5,^16^ providing a means to estimate receptor copy numbers on engineered RBCs.

### HIV-1 enters RBC viral traps

**Figure 2:**
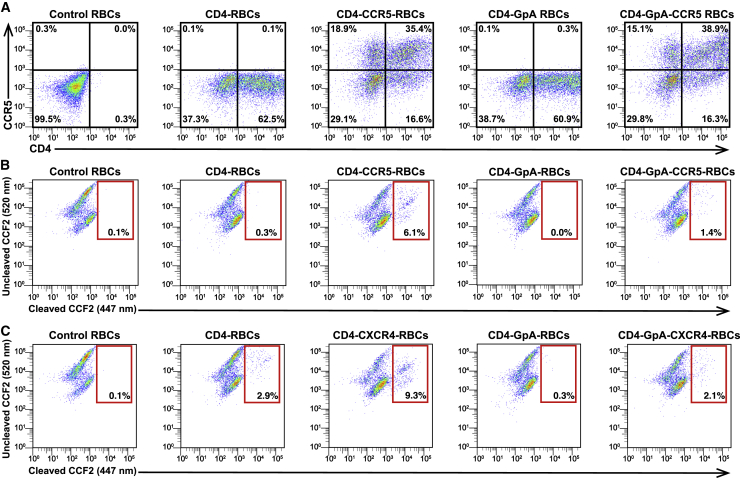
HIV-1 efficiently enters RBC viral traps (A) Flow cytometry measurement of CD4 and CCR5 expression at the end of differentiation for control RBCs, CD4-RBCs, CD4-CCR5-RBCs, CD4-GpA-RBCs, and CD4-GpA-CCR5-RBCs. (B and C) Flow cytometry analysis of HIV-1 infection of engineered RBCs after overnight incubation with a (B) CCR5-tropic HIV-1_YU2_ or (C) CXCR4-tropic HIV-1_HxBc2_ pseudovirus carrying a Vpr-BlaM fusion protein. BlaM cleaves the FRET substrate CCF2-AM in infected cells, resulting in a shift of its emission spectrum from green (520 nm) to blue (447 nm).

To evaluate the efficacy of RBC viral traps against HIV-1, we generated RBCs that expressed CD4 with and without CCR5 or CD4-GpA with and without CCR5 (Figure 2A) and used the β-lactamase (BlaM) fusion assay^17^ to evaluate whether HIV-1 can enter RBC viral traps through attachment of HIV-1 Env spikes to the receptors presented on the RBC surface and subsequent fusion of the viral and RBC membranes. RBCs were incubated with a CCR5-tropic HIV-1_YU2_ pseudovirus carrying a BlaM-Vpr fusion protein that enters cells upon infection. When infected cells are exposed to the fluorescence resonance energy transfer (FRET) substrate CCF2-AM, BlaM cleaves the β-lactam ring in CCF2-AM, resulting in a shift of its emission spectrum from green (520 nm) to blue (447 nm).^17^ Whereas viral entry events were ≤0.3% in control RBCs and CD4-RBCs, entry was detected in 6.1% of CD4-CCR5-RBCs, suggesting that RBC viral traps that present both receptors can entrap the virus. (Figure 2D; Figure S3A). Since only one-third of these RBCs expressed both receptors (Figure 1E), this corresponds to infection of almost 20% of CD4-CCR5-RBCs. CCR5 expression on enucleated RBCs was slightly higher than on nucleated cells; thus, it is unlikely that HIV-1 preferentially entered the small number of remaining nucleated cells (Figure S3B). Higher rates of viral entry were observed for RBCs that co-expressed CD4 and the alternative HIV-1 co-receptor CXCR4 after incubation with a CXCR4-tropic HIV-1 HxBc2 pseudovirus (Figure 2C; Figure S4A). However, lower frequencies of viral entry were detected for RBCs that co-expressed the CD4-GpA fusion protein and CCR5 or CXCR4 (Figures 2B and 2C), and addition of the CD4 D3D4 domains to CD4-GpA did not improve viral entry efficiency (Figure S4B). Unlike CD4, GpA does not localize to lipid raft subdomains,^18^ and thus we speculate that these low rates of viral entry resulted from a lack of co-localization between CD4-GpA and the CCR5 and CXCR4 co-receptors.

### RBC viral traps potently neutralize HIV-1 *in vitro*

**Figure 3:**
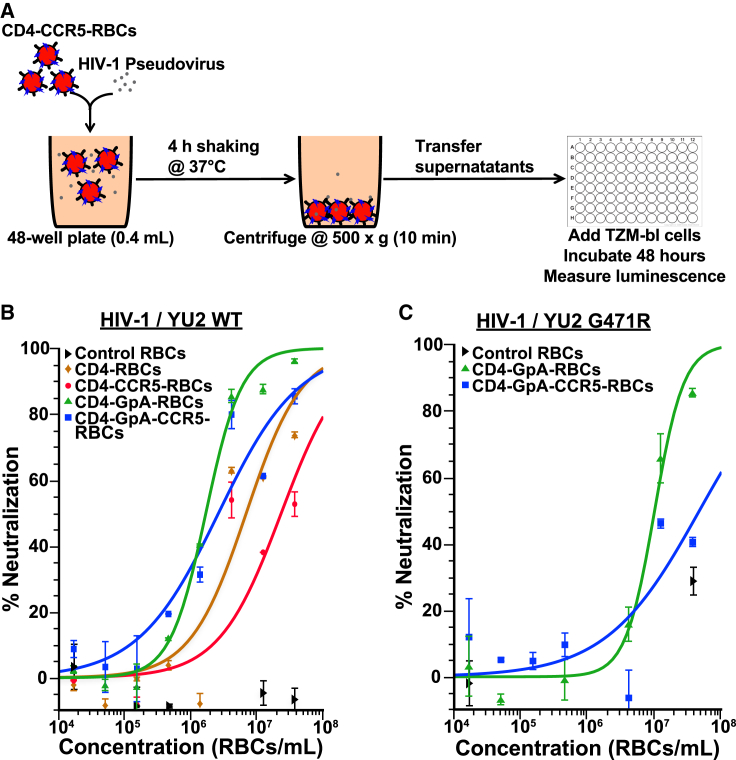
RBC viral traps potently neutralize HIV-1 *in vitro* (A) Schematic illustrating the workflow for the modified neutralization assay used to evaluate the neutralization activity of engineered RBC viral traps. (B) *In vitro* neutralization assay against HIV-1_YU2_ pseudovirus comparing control RBCs (black), CD4-RBCs (brown), CD4-CCR5-RBCs (red), CD4-GpA-RBCs (green), and CD4-GpA-CCR5-RBCs (blue). Data points are the mean and SD of duplicate measurements. (C) *In vitro* neutralization assay against mutant HIV-1_YU2_ Env G471R pseudovirus comparing control RBCs (black), CD4-GpA-RBCs (green), and CD4-GpA-CCR5-RBCs (blue). Data points are the mean and SD of duplicate measurements.

We assessed the therapeutic potential of RBC viral traps using a modified version of the HIV-1 TZM-bl neutralization assay^19^ (Figure 3A). After incubating RBCs with HIV-1_YU2_ pseudovirus, samples were centrifuged to remove RBCs and virions that attached to or infected RBCs. Supernatants containing free virions that had not been captured by RBCs were transferred to 96-well plates and TZM-bl cells were added to measure infectivity. In three independent assays, CD4-GpA-RBCs neutralized HIV-1_YU2_ most potently at an average half-maximal inhibitory concentration (IC_50_) of 1.9 × 10^6^ RBCs/mL (Figure 3B; Table 1). This concentration is equivalent to 0.04% of the RBC concentration of human blood (∼5 × 10^9^ RBCs/mL), suggesting that it would be feasible to achieve therapeutic concentrations *in vivo*. CD4-GpA-RBCs were ∼3-fold more potent than CD4-RBCs, likely due to higher expression levels (Figure 2A; Figure S5). While CCR5 co-expression had no impact on the potency of CD4-GpA-RBCs, co-expression of CCR5 lowered the neutralization activity of CD4-CCR5-RBCs by almost 3-fold in comparison to CD4-RBCs (Figure 3B; Table 1), implying that HIV-1 infection of RBC viral traps was not required for potent neutralization. CCR5 co-expression slightly lowered CD4 expression levels (Figure 2A), potentially explaining the observed drop in potency. However, these results do not exclude the possibility that CCR5 expression on RBC viral traps would have beneficial effects *in vivo*.

**Table 1:** Neutralization potencies of RBC viral traps

Engineered RBCs	IC_50_ (×10^6^ RBCs/mL)				
	Assay 1	Assay 2	Assay 3	Average	SD
Control RBCs	N/D	N/D	N/D	N/D	N/D
CD4-RBCs	7.8	3.3	6.9	6.0	2.2
CD4-CCR5-RBCs	19	5.9	23	16	8.9
CD4-GpA-RBCs	3.0	0.9	1.7	1.9	1.1
CD4-GpA-CCR5-RBCs	3.8	1.4	2.6	2.6	1.2

IC_50_s from three independent *in vitro* neutralization assays from three independent *in vitro* RBC differentiations are shown as ×10^6^ RBCs/mL for control RBCs, CD4-RBCs, CD4-CCR5-RBCs, CD4-GpA-RBCs, and CD4-GpA-CCR5-RBCs. Arithmetic mean IC_50_s and standard deviations (SD) derived from the three experiments are shown. N/D, no data.

We previously showed that virus-like nanoparticles presenting clusters of CD4 (CD4-VLPs) that formed high-avidity interactions with trimeric HIV-1 Env spikes on virions potently neutralized a diverse panel of HIV-1 strains and prevented viral escape *in vitro*.^20^ To confirm that RBC viral traps can also form high-avidity interactions with Env, we evaluated neutralization against a mutant HIV-1_YU2_ Env G471R pseudovirus that was resistant to monomeric soluble CD4, but was sensitive to CD4-VLPs.^20^ CD4-GpA-RBCs potently neutralized the HIV-1_YU2_ G471R pseudovirus (IC_50_ of 1.0 × 10^7^ RBCs/mL) (Figure 3C), suggesting that RBC viral traps and CD4-VLPs would be similarly effective in preventing viral escape through formation of high-avidity interactions with HIV-1 Env spikes.

### RBC viral traps prevent infection of CD4^+^ T cells *in vitro*

**Figure 4:**
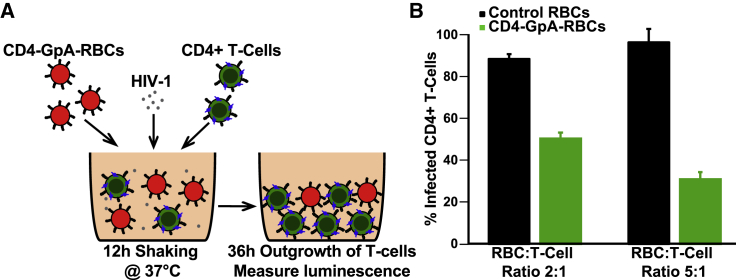
RBC viral traps prevent infection of CD4^+^ T cells *in vitro* (A) Schematic illustrating the workflow for co-incubation of CD4-GpA-RBCs, Rev-A3R5 CD4^+^ T cells, and HIV-1_YU2_ pseudovirus to assess the ability of RBC viral traps to prevent infection of HIV-1 target cells *in vitro*. (B) Bar chart comparing the ability of control RBCs (black) and CD4-GpA-RBCs (green) to reduce the infection rate of Rev-A3R5 CD4^+^ T cells at RBC/T cell ratios or 2:1 (left) and 5:1 (right), respectively.

The ability of RBC viral traps to protect HIV-1 target cells from infection was evaluated by co-culturing control RBCs or CD4-GpA-RBCs with Rev-A3R5 CD4^+^ T cells,^21^ a reporter cell line that expresses luciferase upon HIV-1 infection (Figure 4A). RBCs, CD4^+^ T cells, and HIV-1 pseudovirus were co-incubated at RBC-to-T cell ratios of 2:1 and 5:1 overnight under shaking conditions. The pseudovirus was removed by centrifugation and the cells were re-suspended in Rev-A3R5 CD4^+^ T cell media to permit outgrowth of CD4^+^ T cells. After 36 h, luminescence was measured to determine whether the presence of RBC viral traps prevented infection of CD4^+^ T cells. While control RBCs had no effect, CD4-GpA-RBCs lowered infection rates by 50% and 70%, respectively, demonstrating that RBC viral traps can effectively prevent infection of HIV-1 target cells at RBC/T cell ratios that are ∼1,000-fold lower than typically found in human blood (∼5,000:1)^22^ (Figure 4B). Since HIV-1 did not efficiently enter CD4-GpA-RBCs (Figure 2B), these findings also suggest that high-avidity binding of HIV-1 virions to RBC viral traps is sufficient to prevent attached virions from infecting target cells.

### Erythroblast cell lines stably express viral receptors and continuously produce RBC viral traps against HIV-1 and SARS-CoV-2

**Figure 5:**
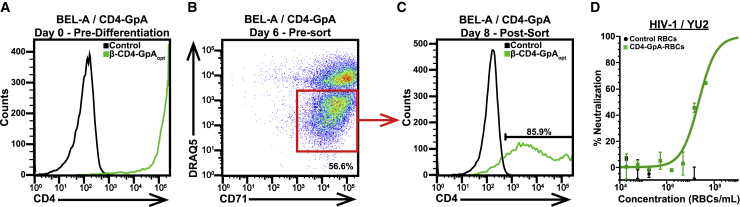
BEL-A erythroblast cell lines stably express CD4-GpA to produce potent RBC viral traps against HIV-1 (A) Flow cytometry measurement of CD4-GpA expression on BEL-A/CD4-GpA cells before differentiation. (B) Flow cytometry analysis of enucleated CD4-GpA-RBCs on day 6 of differentiation. Enucleated RBCs expressed CD71 and did not stain for the nuclear dye DRAQ5. (C) Flow cytometry analysis of CD4-GpA expression on CD71^+^/DRAQ5^−^ BEL-A/CD4-GpA cells post-sorting on day 8 of differentiation. (D) *In vitro* neutralization assay against HIV-1_YU2_ pseudovirus comparing control RBCs (black) and CD4-GpA-RBCs (green). Data points are the mean and SD of duplicate measurements.

To generate a renewable and cost-effective source of RBC viral traps, we engineered the immortalized BEL-A erythroblast cell line^23^ to stably express high levels of CD4-GpA (Figure 5A). The BEL-A/CD4-GpA cells efficiently differentiated into enucleated RBCs, as >50% of CD71-expressing cells did not stain for the nuclear marker DRAQ5 (Figure 5B). After differentiation, CD71^+^/DRAQ5^−^ RBCs were purified using fluorescence-activated cell sorting (FACS). Most RBCs still expressed CD4-GpA (Figure 5C) and potently neutralized HIV-1_YU2_ *in vitro* (IC_50_ of 2.1 × 10^7^ RBCs/mL) (Figure 5D). Independent replicates of *in vitro* differentiation of BEL-A/CD4-GpA cells achieved comparable yields of RBC viral traps (Figures 5B and 5C; Figure S6), suggesting that engineered erythroblast cell lines could be used to continuously produce potent RBC viral traps against HIV-1. However, overall production yields would also depend on the quality of the RBCs, as the viability of BEL-A cells decreases to ∼80% at the end of differentiation^24^ and cells could also get damaged during the purification process. To ensure complete removal of nucleated cells for *in vivo* studies, the RBC viral traps could be further purified using leukoreduction filters and/or gamma irradiation.

**Figure 6:**
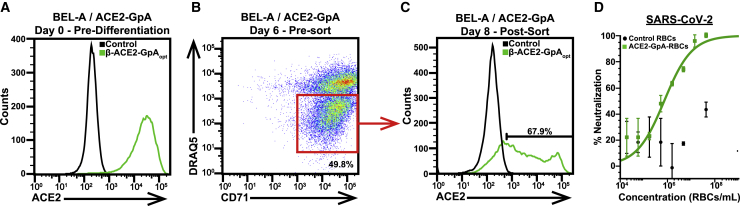
BEL-A erythroblast cell lines stably express ACE2-GpA to produce potent RBC viral traps against SARS-CoV-2 (A) Flow cytometry analysis of ACE2-GpA expression on BEL-A/ACE2-GpA cells before differentiation. (B) Flow cytometry analysis of enucleated ACE2-GpA-RBCs on day 6 of differentiation. Enucleated RBCs expressed CD71 and did not stain for the nuclear dye DRAQ5. (C) Flow cytometry measurement of ACE2-GpA expression on CD71^+^/DRAQ5^−^ BEL-A/ACE2-GpA cells post-sorting on day 8 of differentiation. (D) *In vitro* neutralization assay against lentivirus-based SARS-CoV-2 pseudovirus comparing control RBCs (black) and ACE2-GpA-RBCs (green). Data points are the mean and SD of duplicate measurements.

To evaluate whether RBC viral traps could be effective against other viruses, we generated a BEL-A cell line that continuously produces RBC viral traps against SARS-CoV-2, the virus that caused the ongoing COVID-19 pandemic^25^. BEL-A cells were transduced to stably express a chimeric ACE2-GpA protein containing the extracellular domain of the SARS-CoV-2 receptor ACE2^25^ fused to GpA (Figure 6A). Differentiation efficiency and transgene expression on sorted CD71^+^/DRAQ5^−^ RBCs were comparable to the BEL-A/CD4-GpA cell line (Figures 6B and 6C). Importantly, lentivirus-based SARS-CoV-2 pseudovirus^26^ was highly susceptible to ACE2-GpA-RBC neutralization (IC_50_ of 7 × 10^5^ RBCs/mL) (Figure 6D), suggesting that RBC viral traps have the potential to be effective anti-viral agents against a range of viruses.

## Discussion

In summary, we described engineering strategies that facilitate efficient and continuous production of potent RBC viral traps against HIV-1 and SARS-CoV-2. HIV-1 efficiently entered engineered RBCs expressing HIV-1 receptors, and RBC viral traps potently neutralized the virus *in vitro*, thus demonstrating the desired properties of a viral trap.

A number of techniques have been developed to attach proteins to the RBC surface for therapeutic applications,^27^^,^^28^ including chemical conjugation^29^, ^30^, ^31^, ^32^ and affinity targeting to RBC membrane proteins.^33^, ^34^, ^35^ However, genetic manipulation of RBCs has been challenging due to the loss of cellular organelles during erythroid maturation. RBC surface expression of chimeric proteins containing single-domain antibodies (VHHs) fused to RBC membrane proteins GpA and Kell has been achieved through lentiviral transduction of RBC precursor cells followed by *in vitro* differentiation into reticulocytes.^12^ While shown to be safe and effective in animal models,^12^^,^^27^^,^^28^ all of these methods are limited to RBC surface presentation of soluble or single-pass transmembrane proteins, and important properties of membrane proteins such as localization to specific plasma membrane subdomains and ligand-induced conformational changes and signal transduction activity may not be retained. Moreover, our results showed that fusing the extracellular domain of CD4 to GpA resulted in low surface expression levels in the absence of other optimization steps. However, the combination of an erythroid-specific promoter and transgene codon optimization greatly enhanced CD4-GpA expression and achieved similar expression of wild-type CD4. Importantly, this approach enabled RBC surface expression of the multi-pass transmembrane proteins CCR5 and CXCR4. To the best of our knowledge, this is the first demonstration of robust expression of unmodified non-erythroid transmembrane proteins on the surface of enucleated RBCs. The described engineering strategies could potentially be applied to any type of transmembrane protein and could be widely applicable to genetically engineering therapeutic RBCs.

HIV-1 pseudovirus entered engineered RBCs more efficiently when CCR5 and CXCR4 were co-expressed with wild-type CD4 rather than chimeric CD4-GpA, thus demonstrating that protein modifications that have been used to enhance RBC surface expression^11^^,^^12^ can affect the functionality of the therapeutic protein. A lack of co-localization of CD4-GpA and co-receptors could be the cause of the low entry rates, as CD4 and CCR5 have been shown to co-localize in lipid raft microdomains^13^^,^^14^ and GpA is not typically associated with lipid rafts.^18^ It is also possible that substitution of the membrane-proximal extracellular, transmembrane, or cytoplasmic domains of CD4 interfered with the ability of CD4-GpA to initiate the interaction between HIV-1 Env and co-receptors.

Expression of CD4 or the CD4-GpA fusion protein in the absence of CCR5 was sufficient to potently neutralize HIV-1 *in vitro* due to formation of high-avidity interactions between clusters of CD4 or CD4-GpA on the RBC surface and trimeric HIV-1 Env spikes on virions. RBC viral traps expressing CD4-GpA also reduced HIV-1 infection rates of CD4^+^ T cells, suggesting that viral attachment to RBC viral traps effectively prevents HIV-1 virions from infecting target cells. We previously showed that such high-avidity interactions enhanced the potency of CD4-VLPs by >10,000-fold in comparison to conventional CD4-based inhibitors such as soluble CD4 and CD4-immunoglobulin (Ig), and that HIV-1 was unable to escape against CD4-VLPs *in vitro*.^20^ In contrast to CD4-VLPs that have short *in vivo* half-lives, RBC viral traps could persist *in vivo* for months, implying the RBC approach has the potential to provide sustained control of HIV-1 infection. RBC viral traps neutralized HIV-1 *in vitro* at 2,500-fold lower concentrations than the concentration of total RBCs in human blood and reduced HIV-1 infection of CD4^+^ T cells by 70% at an RBC-to-T cell ratio of 5:1. Given that RBCs outnumber CD4^+^ T cells by ∼5,000:1 in the blood^22^ and CD4^+^ T cell lines are more permissive than natural CD4^+^ T cells,^36^ these results suggest that therapeutic concentrations of RBC viral traps could be achieved *in vivo*.

Erythroblast cell lines that stably express therapeutic proteins represent a renewable and more cost-effective source for large-scale manufacturing of genetically engineered RBCs than CD34^+^ HSCs. BEL-A cell lines that stably expressed CD4-GpA and ACE2-GpA efficiently differentiated into potent RBC viral traps against HIV-1 and the pandemic SARS-CoV-2 virus, respectively, suggesting that RBC viral traps could be effective treatments against a diverse range of viruses. RBC viral traps could become a rapid-response treatment strategy for future viral outbreaks, as erythroblast cell lines could be rapidly developed once a host receptor for a pandemic virus has been identified.

*In vivo* studies will be required to evaluate the safety and efficacy of RBC viral traps, and a number of potential issues need to be addressed. First, it has been shown that reticulocytes generated by *in vitro* differentiation mature *in vivo* into biconcave erythrocytes,^37^ but it needs to be determined whether surface expression of viral receptors is affected by this final maturation step *in vivo*. Second, the half-life of genetically modified RBCs expressing chimeric VHH-GpA/Kell proteins was comparable to control RBCs following intravenous injection in mice,^12^ but it is possible that surface expression of viral receptors would shorten the half-life of RBC viral traps. Third, in the case of CD4 presentation on RBCs, unintended interactions with antigen-presenting cells could have negative implications for the immune system. Fourth, surface presentation of antigens on RBCs has been shown to induce antigen-specific immune tolerance,^38^^,^^39^ so it needs to be investigated whether attachment of viruses to RBC viral traps has detrimental effects on anti-viral immune responses. *In vivo* experiments could address these questions and also elucidate whether entrapment of HIV-1 through co-expression of CCR5 has beneficial effects for viral control and whether entrapped viruses could still infect macrophages following phagocytosis of RBC viral traps. Finally, the ability of genetically engineered RBCs to remove circulating viruses and other pathological agents needs to be compared to other approaches such as nanoparticles coated with cellular membrane^40^ and RBCs modified through conventional techniques.^33^^,^^35^^,^^41^^,^^42^

## Materials and methods

### *In vitro* CD34^+^ HSC differentiation

Human cord blood or mobilized peripheral blood CD34^+^ HSCs (STEMCELL Technologies) were differentiated into enucleated RBCs using a modified version of a previously described protocol.^12^ Briefly, CD34^+^ HSCs were cultured in expansion medium (100 ng/mL recombinant human Flt3 [rhFlt3], 100 ng/mL recombinant human stem cell factor [rhSCF], 20 ng/mL recombinant human interleukin (rhIL)-6, 20 ng/mL rhIL-3, and 100 nM dexamethasone in StemSpan II medium) at a density of 10^5^ cells/mL for 4 days. Cells were then placed in differentiation 1-2 medium (2% human AB plasma, 3% human AB serum, 3 U/mL heparin, 10 ng/mL rhSCF, 1 ng/mL rhIL-3, and 3 U/mL erythropoietin in StemSpan II medium) at a density of 10^5^ cells/mL for 3 days and at 2 × 10^5^ cells/mL for an additional 3 days. The cells were then passaged into differentiation 3 medium (2% human AB plasma, 3% human AB serum, 3 U/mL heparin, 10 ng/mL rhSCF, and 1 U/mL erythropoietin in StemSpan II medium) at a density of 2 × 10^5^ cells/mL for 4 days. To induce RBC maturation, cells were cultured in differentiation 4 medium (2% human AB plasma, 3% human AB serum, 3 U/mL heparin, 0.1 U/mL erythropoietin, and 200 μg/mL holo-transferrin in StemSpan II medium) at a density of 10^6^ cells/mL for 4 days, and in differentiation 5 medium (2% human AB plasma, 3% human AB serum, 3 U/mL heparin, and 200 μg/mL holo-transferrin in StemSpan II medium) at a density of 5 × 10^6^ cells/mL for an additional 3 days. For morphological analysis, cells were spun onto glass slides by cytocentrifugation, stained with May-Grünwald-Giemsa reagents (Sigma-Aldrich), and examined under an LSM 800 laser scanning confocal microscope (Zeiss).

### Transgenes and codon optimization

Human CD4, CCR5, CXCR4, ACE2, and GpA cDNA sequences were obtained from the National Center for Biotechnology Information. The CD4-GpA fusion construct encoded the CD4 signal peptide and D1D2 domains fused to the N terminus of GpA with a nine-residue linker (Glu-Pro-Lys-Thr-Pro-Lys-Pro-Gln-Pro). The ACE2-GpA fusion protein construct encoded the extracellular domain of human ACE2 (residues 1–614) fused to the N terminus of GpA with the nine-residue linker. Transgenes were cloned into the lentiviral backbone plasmids pHAGE-IRES-ZsGreen (PlasmID Repository, Harvard Medical School) for expression under ubiquitous promoters (CMV, EF1α, UBC, and CASI promoters) and pCCL-FB^15^ (provided by Dr. Donald Kohn, UCLA) for erythroid-specific expression. Codon optimization of transgene cDNA sequences was performed using the GeneArt GeneOptimizer software (Thermo Fisher Scientific).

### Lentiviral transduction

VSV-G-pseudotyped lentiviral vectors were produced by co-transfecting HEK293T cells with lentiviral backbone plasmids and packaging plasmids (pHDM-Hgpm2, pHDM-tat1b, pRC/CMV-rev1b, pHDM-G) using FuGENE HD (Promega) according to the manufacturer’s protocol. Supernatants were collected after 48 and 72 h, and lentiviral vectors were concentrated 50-fold using Lenti-X concentrator solution (Takara) according to the manufacturer’s protocol. On day 10 of the differentiation protocol, erythroid progenitor cells were seeded at a density of 10^6^ cells/mL in 12-well plates in the presence of 10 μg/mL Polybrene. 20 μL of concentrated lentiviral vector was added per well and plates were spun for 1.5 h at 850 × *g* at 30°C. Plates were then incubated for 3 h at 37°C before passaging the transduced cells into differentiation 3 medium. For cells that were co-transduced to express two transgenes, 20 μL of each lentiviral vector was added per well. To generate large numbers of engineered RBCs for neutralization assays, two transduction steps were performed on days 10 and 14 of the differentiation protocol.

### Flow cytometry

Transgene expression and RBC maturation efficiency were analyzed by flow cytometry (MACSQuant, Miltenyi Biotec). 2–3 × 10^5^ cells were collected for each condition and samples were stained with the following antibodies: allophycocyanin (APC)-conjugated anti-human CD4 (Invitrogen), fluorescein isothiocyanate (FITC)-conjugated anti-human CD4 (BD Biosciences), FITC-conjugated anti-human CCR5 (BioLegend), phycoerythrin (PE)-conjugated anti-human CXCR4 (Invitrogen), FITC-conjugated anti-human ACE2 (R&D Systems), APC-conjugated anti-CD235ab (BioLegend), and Brilliant Violet 421-conjugated anti-human CD71 (BioLegend). The percentage of enucleated RBCs was measured by double staining cells with APC-conjugated anti-CD235ab and the nuclear stain Hoechst (Thermo Fisher Scientific). Enucleated RBCs were defined as CD235ab^+^/Hoechst^−^ cells. The percentage of enucleated RBCs that expressed transgenes was measured by triple-staining cells with APC-conjugated anti-human CD4, FITC-conjugated anti-human CCR5, and Hoechst nuclear stain.

### BlaM fusion assay

The ability of engineered RBCs to be infected by HIV-1 was evaluated using a modified version of the BlaM assay.^17^ R5-tropic HIV-1_YU2_ and X4-tropic HIV-1_HxBc2_ pseudovirus were produced by co-transfecting a confluent T75 flask of HEK293T cells with the PSG3ΔEnv backbone plasmid (8 μg), the YU2 or HxBc2 Env expression plasmid (4 μg), and a plasmid expressing a BlaM-Vpr fusion protein^43^ (4 μg; provided by Dr. Wesley Sundquist, University of Utah). The supernatant was collected after 72 h and concentrated by centrifugal filtration. 5 × 10^4^ RBCs were seeded in 100 μL of differentiation 5 medium in 96-well plates in the presence of 10 μg/mL Polybrene. 20 μL of concentrated YU2-BlaM-Vpr or HxBc2-BlaM-Vpr pseudovirus was added and plates were spun at 1,000 × *g* for 1 h at 30°C. Plates were then incubated at 37°C overnight. On the next day, freshly prepared 6× CCF2-AM labeling solution was added, and cells were stained for 2 h at room temperature in the dark. After two washes with PBS, the cells were analyzed by flow cytometry (MACSQuant, Miltenyi Biotec).

### HIV-1 neutralization assays

The ability of engineered RBCs to inhibit HIV-1 infection of target cells was tested by using a modified version of the HIV-1 pseudovirus-based TZM-bl assay.^19^ Briefly, serial dilutions of control and engineered RBCs were seeded in 400 μL of TZM-bl media in 48-well plates and incubated with 0.4 μL of HIV-1_YU2_ pseudovirus (50% tissue culture infective dose [TCID_50_] of 3.2 × 10^5^ IU/mL) for 4 h on an orbital shaker (400 rpm) at 37°C in the presence of 10 μg/mL Polybrene. Cells were then spun down at 500 × *g* for 10 min and 155 μL of the supernatants was transferred to 96-well plates. TZM-bl reporter cells (NIH AIDS Reagents Program) were added, and luminescence was measured after 48 h.

### Rev-A3R5 CD4^+^ T cell infection assay

To test whether RBC viral traps can prevent infection of HIV-1 target cells, 10^5^ Rev-A3R5 CD4^+^ T cells^21^ were incubated in 48-well plates with 0.4 μL of HIV-1_YU2_ pseudovirus (TCID_50_ of 3.2 × 10^5^ IU/mL) in 400 μL of TZM-bl media in the presence of 10 μg/mL Polybrene. Control RBCs and CD4-GpA-RBCs were added at RBC/CD4^+^ T cell ratios of 2:1 (2 × 10^5^ RBCs) or 5:1 (5 × 10^5^ RBCs), respectively. Cultures were incubated for 12 h on an orbital shaker (400 rpm) at 37°C overnight. Cells were then spun down at 500 × *g* for 10 min, virus-containing supernatants were removed, and the cells were re-suspended in 400 μL of Rev-A3R5 growth media (RPMI 1640 media supplemented with 10% fetal bovine serum (FBS), 1% penicillin-streptomycin [Pen-Strep], 1% l-glutamine, 1 mg/mL Geneticin, and 1 μg/mL puromycin) to allow outgrowth of CD4^+^ T cells. After 36 h, luminescence was measured for each sample in duplicates and infection rates were calculated as a function of the reduction in average luminescence compared to the control infection of Rev-A3R5 CD4^+^ T cells in the absence of RBCs.

### SARS-CoV-2 neutralization assays

Lentivirus-based SARS-CoV-2 pseudovirus was generated by transfecting HEK293T cells with a luciferase-expressing lentiviral backbone plasmid, accessory plasmids (pHDM-Hgpm2, pHDM-tat1b, pRC/CMV-rev1b), and a plasmid encoding the SARS-CoV-2 spike protein with a 21-residue cytoplasmic tail deletion (Wuhan Hu-1 strain; GenBank: NC_045512). The neutralization activity of ACE2-GpA RBCs was measured using a modified version of a recently reported protocol.^26^ 1.25 × 10^4^ 293T-ACE2 cells (provided by Dr. Jesse Bloom, Fred Hutchinson Cancer Research Center) were seeded per well on poly-l-lysine-coated 96-well plates (Corning Life Sciences) 18 h before infection. Serial dilutions of control and ACE2-GpA-RBCs were seeded in 400 μL of media (DMEM supplemented with 10% FBS and Pen-Strep) and incubated with 3 μL of lentiviral particles pseudotyped with the SARS-CoV-2 spike protein (5.5 × 10^7^ relative light units [RLU]/mL) for 4 h on an orbital shaker (400 rpm) at 37°C in the presence of 10 μg/mL Polybrene. The lentiviral backbone of this SARS-CoV-2 pseudovirus system expresses luciferase to enable detection of infected cells. RBCs were spun down at 500 × *g* for 10 min and 100 μL of supernatant was transferred to the 96-well plate with the seeded 293T-ACE2 cells. Luminescence was measured after 48 h using a plate reader (Tecan).

### Generation of stable erythroblast cell lines

Immortalized BEL-A erythroblast cells^23^ (provided by Dr. Jan Frayne, University of Bristol) were transduced with VSV-G-pseudotyped lentiviral vectors carrying the CD4-GpA or ACE2-GpA transgenes in the erythroid-specific pCCL-FB expression cassette. To allow positive selection of cells that stably expressed the transgenes, the puromycin-*N*-acetyltransferase gene was added downstream of the transgene and a P2A cleavage peptide.^44^ Stable BEL-A/CD4-GpA and BEL-A/ACE2-GpA cell lines were generated by growing the transduced cells in expansion media (50 ng/mL rhSCF, 3 U/mL erythropoietin, 1 μM dexamethasone, and 1 μg/mL doxycycline in StemSpan II medium) in the presence of 0.25 μg/mL puromycin for 3–4 weeks. Differentiation of BEL-A cells was initiated as described^23^ by transferring the cells into primary media (3% human AB serum, 2% FBS, 3 U/mL heparin, 10 ng/mL rhSCF, 1 ng/mL rhIL-3, 3 U/mL erythropoietin, 200 μg/mL holo-transferrin, and 1 μg/mL doxycycline in StemSpan II medium) for 3–4 days at a density of 2 × 10^5^ cells/mL. To induce RBC maturation, cells were moved into tertiary media (3% human AB serum, 2% FBS, 3 U/mL heparin, 3 U/mL erythropoietin, 500 μg/mL holo-transferrin, and 1 U/mL Pen-Strep in StemSpan II medium) for 4 days at a density of 1 × 10^6^ cells/mL.

### FACS

Enucleated RBCs were purified by FACS on day 7 of the BEL-A differentiation protocol. Brilliant Violet 421-conjugated anti-human CD71 antibody (BioLegend) and the nuclear stain DRAQ5 (Abcam) were diluted 1:100 and 1:1,000 in PBS+ (PBS supplemented with 2% FBS). Cells were stained at a concentration of 2.5 × 10^7^ cells/mL for 30 min at room temperature in the dark. After two washes in PBS+, cells were resuspended in PBS+ at a concentration of 1 × 10^7^ cells/mL. Enucleated RBCs were defined as CD71^+^/DRAQ5^−^ cells and this cell population was purified using a SONY SH800 cell sorter (Sony Biotechnology).

## Supplemental information

Document S1. Figures S1–S6

Document S2. Article plus supplemental information
