# Supplementary material for: In vitro characterization of engineered red blood cells as viral traps against HIV-1 and SARS-CoV-2
Source: Mol Ther Methods Clin Dev. 2021 Mar 10;21:161–70. doi: 10.1016/j.omtm.2021.03.003 (PMC7944778; doi:10.1016/j.omtm.2021.03.003)
Supplement: Document S1. Figures S1–S6 [file mmc1.pdf]

OMTM, Volume *21*

Supplemental information

In vitro characterization of engineered red blood cells as viral traps against HIV-1 and SARS-CoV-2

Magnus A.G. Hoffmann, Collin Kieffer, and Pamela J. Bjorkman

Supplemental Information

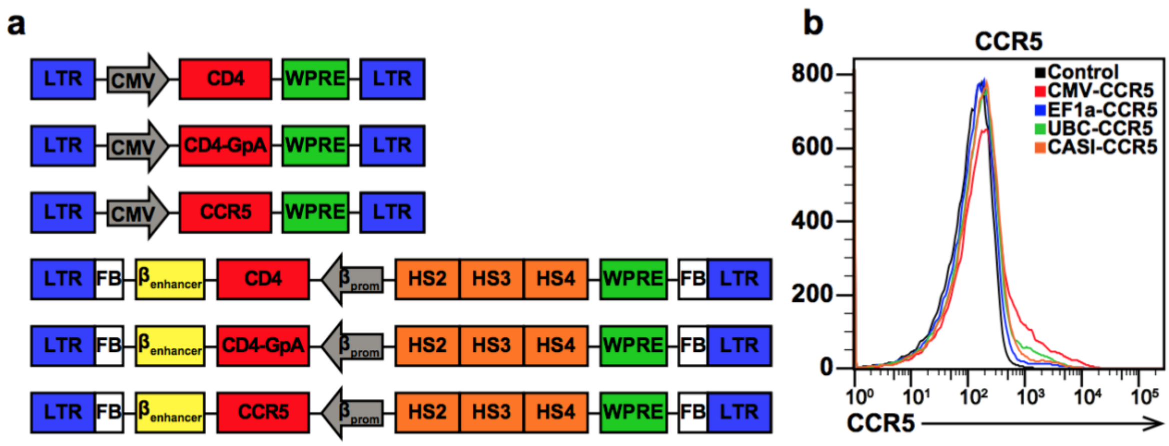

Fig. S1 Lentiviral vector constructs for engineering RBCs. a, Schematic of pHAGE-based and pCCL-FB-based lentiviral vector constructs used for the delivery of CD4, CD4-GpA, and CCR5 transgenes. **b**, Comparison of ubiquitous CMV, EF1-α, UBC, and CASI promoters for the expression of CCR5 in erythroid progenitor cells on day 13 of differentiation.

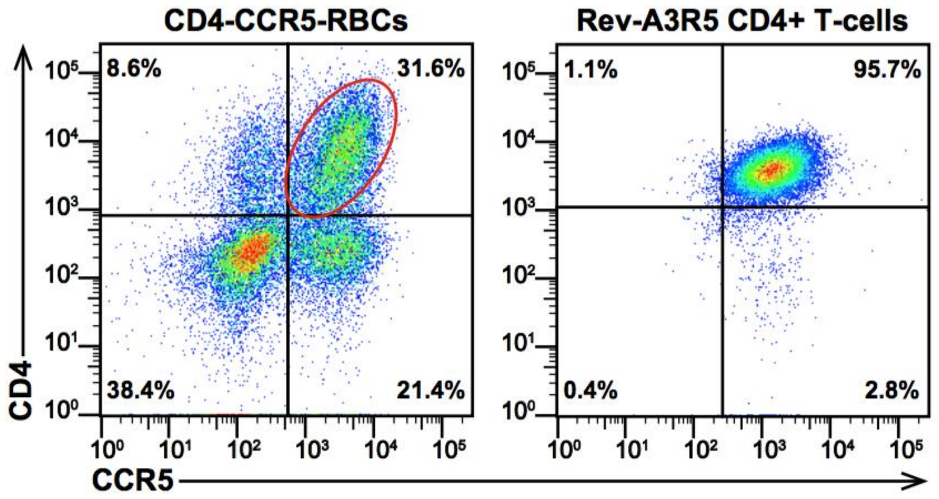

Fig. S2 Comparison of HIV-1 receptor expression levels on CD4-CCR5-RBCs and CD4+ T-cells. Flow cytometry analysis of CD4 and CCR5 expression on enucleated CD4-CCR5-RBCs and Rev-A3R5 CD4+ T-cells.

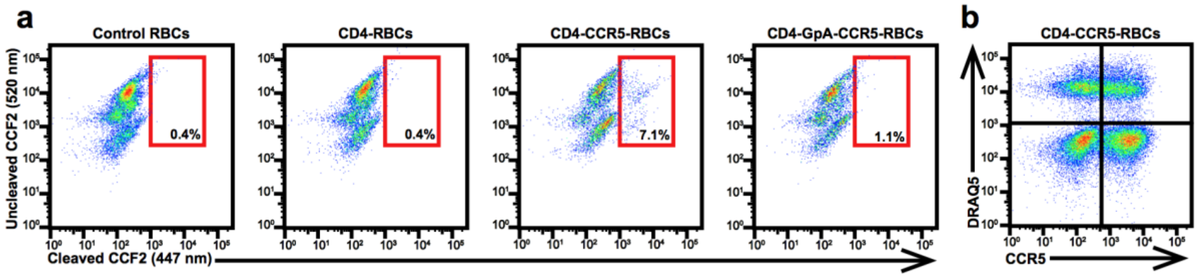

Fig. S3 BlaM assay reproducibly shows that HIV-1 enters RBC viral traps. a, Flow cytometry analysis of HIV-1 infection of engineered RBCs after overnight incubation with a CCR5-tropic HIV-1$_{YU2}$ pseudovirus carrying a Vpr-BlaM fusion protein. These results represent an independent biological replicate of the experiment shown in Fig. 2b using engineered RBCs from a different in vitro differentiation culture. **b**, Comparison of CCR5 expression on enucleated (DRAQ5-) and nucleated (DRAQ5+) CD4-CCR5-RBCs.

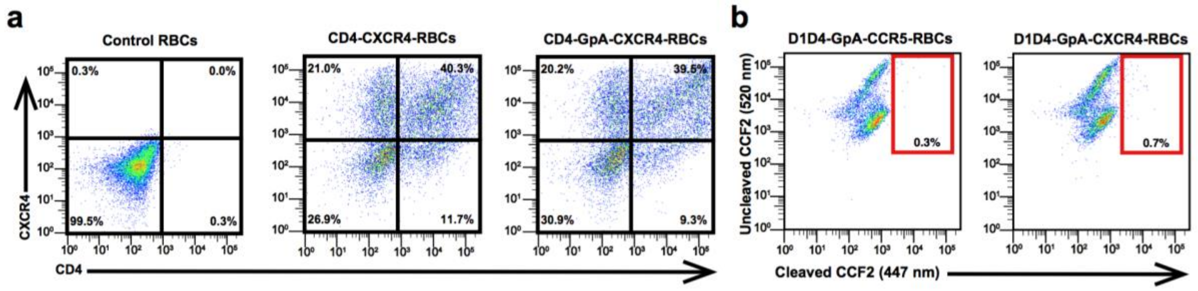

Fig. S4 Engineered RBCs express CD4 and CXCR4 and can be infected by X4-tropic HIV-1. a, Flow cytometry measurement of CD4 and CXCR4 expression at the end of differentiation for control RBCs, CD4-CXCR4-RBCs, and CD4-GpA-CXCR4-RBCs. **b**, Flow cytometry analysis of HIV-1 infection of RBCs expressing a chimeric D1D4-GpA fusion protein that contained the CD4 D1D4 domains to evaluate if addition of the CD4 D3D4 domains enhanced infection. BlaM assays were performed with R5-tropic HIV-1_{YU2} and X4-tropic HIV-1_{HxBc2} pseudovirus on D1D4-GpA-CCR5-RBCs (left) and D1D4-GpA-CXCR4-RBCs (right), respectively.

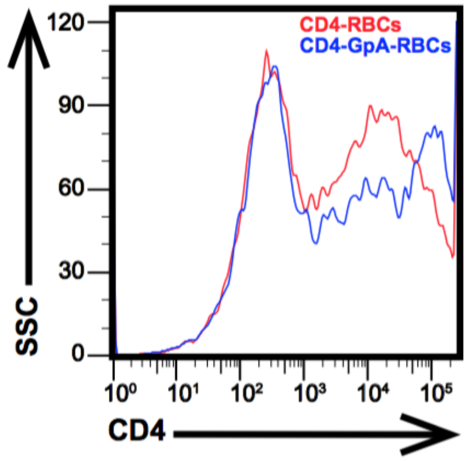

Fig. S5 Comparison of expression levels between CD4-RBCs and CD4-GpA-RBCs. Flow cytometry analysis of CD4 and CD4-GpA expression levels on CD4-RBCs and CD4-GpA-RBCs, respectively, at the end of differentiation.

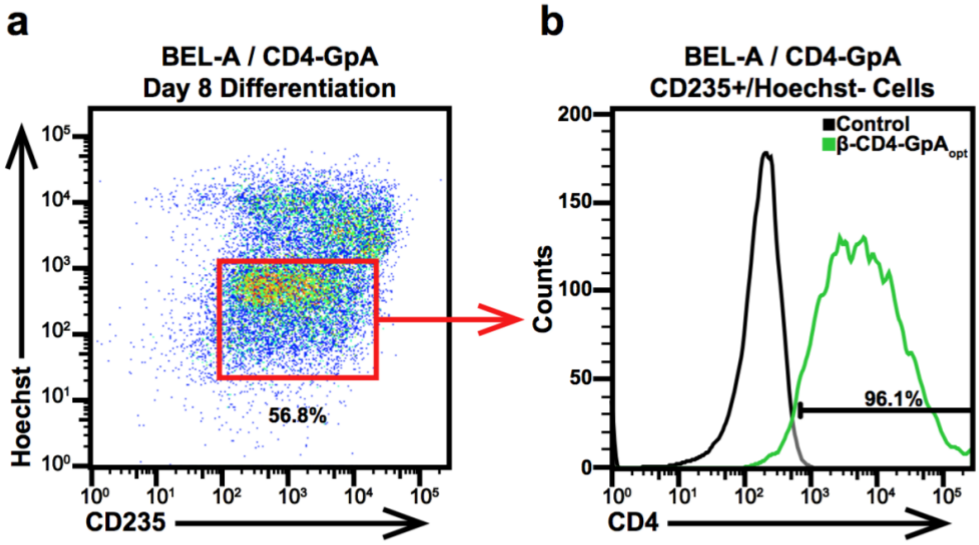

Fig. S6 In vitro differentiation of BEL-A / CD4-GpA cells consistently produces RBC viral traps. a, Flow cytometry analysis of enucleated CD4-GpA-RBCs on day 8 of in vitro differentiation. Enucleated RBCs expressed CD235 and did not stain for the nuclear dye Hoechst. **b**, Flow cytometry analysis of CD4-GpA expression on CD235+/Hoechst- BEL-A / CD4-GpA cells. These results represent an independent biological replicate of the experiment shown in Fig. 5.
